# Supplementary material for: Theoretically Revealing the Response of Intermolecular Vibration Energy Transfer and Decomposition Process of the DNTF System to Electric Fields Using Two-Dimensional Infrared Spectra
Source: Int J Mol Sci. 2023 Feb 22;24(5):4352. doi: 10.3390/ijms24054352 (PMC10002173; doi:10.3390/ijms24054352)
Supplement: Supplementary file 1 [file ijms-24-04352-s001.zip › ijms-2121333-supplementary.pdf]

Supporting Information

Theoretically Revealing the Response of Intermolecular Vibration Energy Transfer and Decomposition Process of the DNTF System to Electric Fields using Two-dimensional Infrared Spectra

Hai-Chao Ren[1], Lin-Xiang Ji[2], Xian-Zhen Jia[1,] Jun Tao[1], Rui-Peng Liu[1],

Dong-Qing Wei[3,4], Xiao-Feng Wang[1, *] and Guang-Fu Ji[5,*]

[1]Xi'an Modern Chemistry Research Institute, Xi'an 710065, China

[2]Department of Physics and Engineering Physics, University of Saskatchewan, Saskatoon, Saskatchewan, S7N5E2, Canada

[3]College of Food Science and Engineering, Henan University of Technology, Zhengzhou, 450001, China

[4]College of Life Science and Biotechnology, Shanghai Jiao Tong University, Shanghai 200240, China

[5]National Key Laboratory for Shock Wave and Detonation Physics Research, Institute of Fluid Physics, Chinese Academy of Engineering Physics, Mianyang 621000, China

[*] Corresponding Author Emails: wangxf204@163.com; cyfjkf@caep.cn

Table S1. Coordinates (Å) of DNTF primitive cell shown in Figure 1.

Element	X	Y	Z	Element	X	Y	Z
O	-0.9604	1.8687	1.2108	O	-9.9726	-0.7683	-0.4091
O	-2.7101	-2.6912	-1.8012	O	-4.8496	1.4700	0.8863
O	1.0904	-3.0534	2.2252	O	-5.4362	-3.7408	-0.9579
O	-0.8054	0.6246	-2.9511	O	-8.3203	0.9526	3.1511
O	-0.5288	2.7684	-2.6657	O	-10.2848	1.8096	2.7249
O	-3.0243	-1.6995	2.3356	O	-6.4735	0.1167	-2.6773
O	-1.9195	-1.1366	4.1324	O	-6.6885	-1.5308	-4.0916
O	-3.2849	-0.5461	-2.4165	O	-6.8164	2.5418	1.4317
N	-1.4288	0.5874	1.1248	N	-8.6241	-0.6617	-0.6135
N	-0.7200	2.4009	-0.0066	N	-10.3757	-0.0772	0.6863
N	-2.6437	-1.2310	-1.6573	N	-6.3141	1.5494	0.9795
N	-1.9702	-3.2533	-0.8161	N	-4.5271	0.2930	0.3022
N	0.5801	-3.0509	0.9500	N	-5.2932	-2.7528	-0.0144
N	0.2262	-2.5148	3.1216	N	-5.8657	-3.2528	-2.1482
N	-0.7748	1.6404	-2.2790	N	-9.3074	1.1455	2.4587
N	-2.0175	-1.6166	3.0262	N	-6.4244	-1.0605	-3.0079
C	-1.0254	1.4628	-0.8525	C	-9.2918	0.4428	1.1742
C	-1.4772	0.3055	-0.1520	C	-8.1707	0.0854	0.3636
C	-1.8554	-1.0106	-0.6109	C	-6.7675	0.3974	0.4764
C	-1.4728	-2.2837	-0.1046	C	-5.6330	-0.3427	0.0484
C	-0.6071	-2.5198	1.0508	C	-5.6376	-1.6482	-0.6165
C	-0.8135	-2.1840	2.4198	C	-5.9937	-1.9770	-1.9554
O	5.9634	2.7483	-1.0323	O	4.3480	-3.6337	1.6048
O	1.8513	2.6646	2.9904	O	6.6956	-0.2125	-2.3802
O	1.6420	6.2767	-1.1449	O	1.4365	-1.8725	-2.7847
O	6.4555	1.6300	3.0207	O	7.9551	-3.8800	-0.6476
O	6.3814	-0.1854	1.8102	O	8.4150	-4.0178	1.4844
O	2.1340	1.9752	-1.3485	O	3.5635	0.0516	0.4702
O	2.5532	2.9552	-3.2458	O	1.4213	-0.0463	0.8014
O	3.6971	1.3106	3.1997	O	7.9655	-1.1323	-0.6872
N	4.7677	3.1700	-0.5306	N	4.2358	-2.6914	0.6212
N	6.5921	1.9040	-0.1719	N	5.6220	-4.0848	1.7258
N	3.1545	2.1618	2.5484	N	6.8846	-1.0829	-1.2020
N	1.4065	3.5552	2.0804	N	5.3963	-0.2554	-2.7376

N	1.8183	5.7931	0.1288	N	2.7847	-1.8630	-3.0547
N	1.8046	5.3154	-2.0831	N	1.1567	-1.3165	-1.5851
N	6.2443	1.0091	2.0008	N	7.6871	-3.8017	0.5414
N	2.2751	2.9632	-2.0743	N	2.4294	-0.2735	0.1601
C	5.8080	1.8141	0.8540	C	6.2951	-3.4453	0.8207
C	4.6333	2.6089	0.6470	C	5.4348	-2.5515	0.1110
C	3.4283	2.8103	1.4114	C	5.6856	-1.6234	-0.9629
C	2.3038	3.6468	1.1397	C	4.7880	-1.0658	-1.9175
C	2.0788	4.5223	-0.0118	C	3.3458	-1.2982	-2.0207
C	2.0696	4.2395	-1.4097	C	2.3074	-0.9646	-1.1057

Table S2. Theoretical bond length (Å) of DNTF when E-Field is -2.0568 V/nm.

Bond	DNTFI	DNTFII	DNTFIII	DNTFIV
N7-O5	1.212	1.221	1.219	1.218
N7-O6	1.218	1.208	1.216	1.215
C1-N7	1.467	1.467	1.460	1.464
C1-C2	1.432	1.430	1.426	1.431
C2-N2	1.311	1.311	1.308	1.312
O1-N2	1.362	1.369	1.366	1.370
O1-N1	1.359	1.359	1.351	1.357
N1-C1	1.295	1.296	1.299	1.297
C2-C3	1.443	1.441	1.446	1.439
C3-N3	1.337	1.339	1.326	1.336
N3-O4	1.199	1.192	1.212	1.205
O2-N3	1.475	1.490	1.464	1.458
O2-N4	1.348	1.343	1.357	1.353
N4-C4	1.304	1.304	1.301	1.300
C3-C4	1.427	1.423	1.424	1.422
C4-C5	1.464	1.464	1.462	1.465
C5-C6	1.426	1.425	1.423	1.423
C6-N5	1.297	1.296	1.298	1.296
N5-O3	1.353	1.353	1.353	1.356
O3-N6	1.375	1.377	1.372	1.373
N6-C5	1.304	1.305	1.304	1.304
C6-N8	1.454	1.447	1.459	1.459
N8-O7	1.235	1.215	1.227	1.224
N8-O8	1.204	1.221	1.209	1.211

Table S3. Theoretical bond length (Å) of DNTF when E-Field is -1.0284 V/nm.

Bond	DNTFI	DNTFII	DNTFIII	DNTFIV
N7-O5	1.212	1.209	1.219	1.213
N7-O6	1.218	1.221	1.217	1.219
C1-N7	1.467	1.465	1.460	1.464
C1-C2	1.433	1.430	1.426	1.430
C2-N2	1.311	1.311	1.308	1.311
O1-N2	1.363	1.368	1.367	1.369
O1-N1	1.359	1.358	1.350	1.357
N1-C1	1.294	1.296	1.299	1.297
C2-C3	1.442	1.441	1.445	1.441
C3-N3	1.337	1.338	1.327	1.336
N3-O4	1.200	1.195	1.209	1.203
O2-N3	1.470	1.483	1.466	1.464
O2-N4	1.348	1.346	1.356	1.353
N4-C4	1.303	1.304	1.301	1.300
C3-C4	1.427	1.423	1.423	1.421
C4-C5	1.464	1.464	1.463	1.465
C5-C6	1.426	1.424	1.424	1.424
C6-N5	1.297	1.295	1.298	1.297
N5-O3	1.353	1.352	1.355	1.356
O3-N6	1.374	1.376	1.373	1.373
N6-C5	1.305	1.305	1.304	1.304
C6-N8	1.454	1.447	1.461	1.460
N8-O7	1.234	1.218	1.210	1.211
N8-O8	1.204	1.218	1.225	1.224

Table S4. Theoretical bond length (Å) of DNTF when E-Field is 1.0284 V/nm.

Bond	DNTFI	DNTFII	DNTFIII	DNTFIV
N7-O5	1.217	1.212	1.218	1.209
N7-O6	1.213	1.221	1.219	1.223
C1-N7	1.468	1.462	1.458	1.464
C1-C2	1.434	1.429	1.426	1.428
C2-N2	1.312	1.311	1.309	1.311
O1-N2	1.364	1.366	1.367	1.367
O1-N1	1.360	1.356	1.350	1.356
N1-C1	1.294	1.297	1.300	1.298
C2-C3	1.440	1.441	1.443	1.443
C3-N3	1.337	1.336	1.330	1.336
N3-O4	1.203	1.201	1.205	1.199
O2-N3	1.460	1.471	1.471	1.476
O2-N4	1.349	1.351	1.353	1.353
N4-C4	1.303	1.303	1.301	1.301
C3-C4	1.428	1.425	1.422	1.420
C4-C5	1.464	1.464	1.463	1.465
C5-C6	1.426	1.423	1.425	1.424
C6-N5	1.297	1.295	1.297	1.297
N5-O3	1.353	1.351	1.358	1.357
O3-N6	1.373	1.374	1.375	1.374
N6-C5	1.305	1.295	1.305	1.304
C6-N8	1.453	1.447	1.464	1.462
N8-O7	1.234	1.222	1.222	1.224
N8-O8	1.204	1.214	1.210	1.210

Table S5. Theoretical bond length (Å) of DNTF when E-Field is 2.0568 V/nm.

Bond	DNTFI	DNTFII	DNTFIII	DNTFIV
N7-O5	1.213	1.220	1.216	1.225
N7-O6	1.218	1.215	1.221	1.208
C1-N7	1.469	1.461	1.457	1.465
C1-C2	1.435	1.429	1.426	1.427
C2-N2	1.312	1.310	1.309	1.310
O1-N2	1.362	1.366	1.368	1.366
O1-N1	1.359	1.356	1.351	1.355
N1-C1	1.295	1.297	1.299	1.298
C2-C3	1.439	1.441	1.439	1.445
C3-N3	1.336	1.334	1.331	1.336
N3-O4	1.205	1.205	1.205	1.197
O2-N3	1.455	1.465	1.469	1.482
O2-N4	1.350	1.355	1.353	1.354
N4-C4	1.303	1.302	1.301	1.302
C3-C4	1.429	1.425	1.421	1.420
C4-C5	1.463	1.465	1.463	1.465
C5-C6	1.426	1.422	1.426	1.424
C6-N5	1.296	1.296	1.297	1.296
N5-O3	1.352	1.350	1.359	1.357
O3-N6	1.372	1.374	1.375	1.375
N6-C5	1.305	1.304	1.305	1.304
C6-N8	1.454	1.448	1.465	1.462
N8-O7	1.236	1.225	1.222	1.224
N8-O8	1.204	1.213	1.210	1.209

Table S6. Comparison of calculated (ω_{cal}, cm^{-1}) and experimental (ω_{exp}, cm^{-1}) infrared shifts [1–4] and vibrational mode assignments of DNTF.

$\omega_{cal.}$	$\omega_{exp.}$	Description
1579.7	1584.0	$N7O_2$ asymmetric stretching and furuzan vibration in DNTFI, $N8O_2$ asymmetric stretching in DNTFⅡ
1603.3		$N7O_2$ asymmetric stretching in DNTFIV
1612.8	1612.0	$N8O_2$ asymmetric stretching and furuzan vibration in DNTFII
1620.0		$N7O_2$ asymmetric stretching in DNTFII
1622.4		$N8O_2$ asymmetric stretching and furuzan vibration in DNTFIV
1623.2		$N7O_2$ asymmetric stretching and furuzan vibration in DNTFIII, furuzan ring vibration in DNTFⅣ
1625.9		$N8O_2$ asymmetric stretching and furuzan vibration in DNTFIII, $N8O_2$ asymmetric stretching and furuzan vibration in DNTFⅣ
1631.6		$N8O_2$ asymmetric stretching and furuzan vibration in DNTFI
1640.8	1640.0	$N8O_2$ asymmetric stretching in DNTFIV
1655.2		$N7O_2$ asymmetric stretching and furuzan vibration in DNTFIII
1659.8		$N8O_2$ asymmetric stretching and furuzan vibration in DNTFII, $N7O_2$ asymmetric stretching in DNTF Ⅰ
1682.5		$N8O_2$ asymmetric stretching and furuzan vibration in DNTFI, $N7O_2$ asymmetric stretching in DNTFⅣ
1688.4		$N8O_2$ asymmetric stretching in DNTFIII

Note: abbreviation used here is as follows: as = asymmetric stretching vibration

Reference

1. Zhou, Y.; Zhang, Z.; Li, J.; Guan, X.; Huang , X.; Zhou, C. Crystal Structure of 3,4-dinitrofurazanofuroxan. *Chin. J. Explos. Propell.* **2005,** 28(2), 43-52.

2. Sheremerev, A. V.; Ivanova, E. A.; Spiridonova, N. P.; Melnikova, S. F.; Antipin, M. Y. Desilylative nitration of C,N-disilylated 3-amino-4-methylfurazan. *J. Heterocycl. Chem.* **2005,** 42, 1237.

3. Ma, H. –X.; Song J. –R.; Xiao, H. –M.; Hu, R. –Z.; Zhao, F. –Q. Density Functional Theoretical Investigation on 3, 4-Dinitrofurazanfuroxan (DNTF). *Chin. J. Explos. Propell.* **2006,** 3, 43.

4. Li, Y.; Yuan, J.; Zhao, W.; Qu, Y.; Xing, X.; Meng, J.; Liu, Y. Application and Development of 3, 4-Bis (3-nitrofurazan-4-yl) furoxan (DNTF). *Russ. J. Gen. Chem.* **2021,** 91 (3), 445-455.
